# Institutes of Dental Science

**Published:** 1863-08

**Authors:** W. H. Atkinson


### INSTITUTES OF DENTAL SCIENCE.

(Read before the American Dental Convention.)

BY W. H. ATKINSON, M. D.

In dentistry, as in all else that involves a community of knowledge and practice, really representative men are extremely scarce. The immense aggregations of basal principles of seemingly remote and disconnected departments of science, which focalize in the principles and practice of dentistry actually constitute the whole range of mechanics, physics and metaphysics. So that he who shall truly represent the institutes of dental science must be the embodiment of fact and philosophy throughout the domain of organic living forces and the unseen, so-called, laws of dynamics.

In view of this statement is it not evident that we need an entirely new revelation of textual teachings, taken from all the departments of anatomy with its sequential sciences, of physiology, pathology, chemistry, &c., &c.?

We are told by the wiseacres of our body that there is abundance already upon the record to afford us all the light necessary to a scientific rendering of service in answering the queries and needs of our patients and those who desire to be instructed how they may avoid the necessity of demanding our practical services.

But these same boastedly well-posted, dignified asserters of the abundance of recorded answers to these important enquiries are as lame as the more humble among us when put to the test of proving what they assert with such confidence.

When such men as Todd, Bowman, Tomes, Brown Sequard, Rokistansky and all the great lights in physical science deign to revise and modify their best works even to mutilation to such degree as to very much reduce the value of their former writings and labors, what shall we not say of the crudities that have formerly passed for institutes to guide us in our enhanced and arduous scientific professional labors? Much as we owe to Hunter, Bell, Fox, &c., &c., if we were to tarry in such doctrines as they advocated and dubb them institutes, we should cut but a sorry figure in our attempts to stand in the front ranks of the teachers and practitioners of the present time. The assertion made by some of these men of the *in*organic character of the teeth and by others of the capability of normal dentine to suppurate do not very well accord together nor yet with the truth! But when viewed in the light of new observations made under more advantageous circumstances we may readily conceive how they fell into the errors which they recorded leading so many away from the line of correct observation and beneficent practice that now begins to prevail in the surgery of the mouth!

The institutes of dental science proper demand the aphoristic textual statement of all science specially pointed to its outcrop in the creation, development, sickening and restoration of the teeth; as basis and guide to an intelligent and successful supervision and the performance of the necessary manipulations to secure or render them sound and efficient in the performances of the functions assigned them in the animal economy.

He who assumes to teach this department of dental science entitles us to look to him for the solution, in principle, of all the perplexities which are liable to arise in the whole range of operative and mechanical dentistry! In view, then, of the immense responsibility attached to this chair, who is there among us who may not well shrink from assuming the performance of the duties legitimately arising within its least extended domain?

Notwithstanding the lively appreciation with which I regard the responsibilities of this department of instruction without which all others are useless, I heartily rejoice that just such an onerous chair has been inaugurated in at least two dental schools.

The sense of need for such a chair is sure to bring out or create the ability to fill it just so soon as the high necessity is generally felt and acknowledged in the profession. I say acknowledged, and by that I do not mean a tacit assent of the mind merely, but a hearty expression of that conviction on all occasions coupled with earnest endeavor to add some mite to the general interest by inquiry or a showing how and where to apply some principle which constitutes a focal point of demonstration indicative of its relation to and control of correct practice in the premises, which by multiplication and aggregation will soon become the beginning of a code of principles or institutes capable of improvement and enlargement in the ratio of the numbers thus actively engaged in preparing a reliable guide to coming inquirers for a plain path in which to walk.

To be sure "theory" and "principles" usually coupled with "practice" in medical schools means the same thing but does not express the real function of the chair in such manly unequivical terms!

Although, as hinted, it smacks of impudence to accept such a chair, it also indicates a very different quality of mind, viz: a willingness to try to be of service in a direction that the nomination to the chair indicated ability was thought to lay, which of itself is earnest of the fact as has been often proved in the calling of men to posts they deemed themselves utterly unfit to fill, proving to be our very first teachers and leaders so soon as they had time to bring their latent powers into the work.

There is a possibility of a man's having fitness for a post and at the same time being the first to perceive it, but it is not the *rule* in military, civil or literary life; and hence I should prefer to have others perceive the adaptation and act thereupon, rather than to have the aspirant nominate himself and like a politician busy himself in securing the election that it were quite possible he might disgrace.

There is a quality and range of mind which, naturally ethical, as naturally thirsts to see principles dominate practice, as they should, as does the practical, barely fact mind demand that practice shall be the touchstone for all theory which at best is regarded as of but little account.

He who feels that he has a mission to fulfill must needs be about it. And he will often be quite unconscious of any peculiar fitness for that special department other than the abiding desire that higher culture prevailed in that direction. Such a mind will teach irrespective of consequences, simply because he must; by continuance of which course he must be more than ordinarily dull if he do not become transcendantly fitted for the course sedulously followed under the spontaneity of an ardent nature. Much as I adore the high status necessary to an honorable filling of a chair of institutes, and much as I deplore the wide-spread ignorance characteristic of our apparent status as a profession, I have no doubt that the latent ability is now embodied among us respectably to fill not only two but twenty chairs of this elevated character. All that is necessary is to find them out, which can be done in no other way so well as by a free and fraternal intercourse with every man in our whole ranks.

For so soon as true freedom and fraternity shall prevail we shall know each other and ourselves as we never can so long as we stand aloof from either freedom or fraternity, the genuine exercise of which will soon metamorphose us from items of isolated knowledge and skill into a compact body of men and principles whose labors and coalescence shall constitute a system of institutes that the greatest may well be proud to find himself able to teach!

A correct understanding of the institutes of dental science can alone fortify us against surprises and discomfiture in new and anamalous cases arising in the practice of dentistry.

It may be said that we owe much of our institutes to the blunders and mal-practices of the former times, and therefore each can work out at the last his own theories which govern him in practice; to which I would reply that before we had a profession this was to some degree necessary. But I regard it as akin to the folly of the farmer's son who was raised in a densely and heavily wooded region learning the necessity there of clearing off the timber first and plowing and sowing after; having moved to the prairie country of the west insisted upon planting and growing trees all over that wide expanse to prepare them for the process of clearing that the good old way of farm making might suffer no innovations.

How many generations would die out before the maturity of his trees? And how long would the world have to wait for an enlightened practice if we were to depend upon the isolated work and knowledge of individuals to teach the institutes of dental science without association and without organized institutions for the investigation and diffusion of the knowledge of the laws, or institutes, upon which all intelligent correct procedure depends?

Let us take, then, the rich prairie ground of the profession as it is, and plow deep and settle thickly within easy reach of each other that our work may be properly apportioned, so that the greatest facilities in teaching and practice in the various branches and departments of acquiring and performing may become established institutions among us to the joy and satisfaction of each and of all!

